# Targeting epidermal fatty acid binding protein for treatment of experimental autoimmune encephalomyelitis

**DOI:** 10.1186/s12865-015-0091-2

**Published:** 2015-05-12

**Authors:** Enyu Rao, Puja Singh, Yan Li, Yuwen Zhang, Young-In Chi, Jill Suttles, Bing Li

**Affiliations:** The Hormel Institute, University of Minnesota, 801 16th Avenue, NE, Austin, MN 55912 USA; Department of Microbiology and Immunology, University of Louisville, 319 Abraham Flexner Way, Louisville, KY 40202 USA

**Keywords:** Fatty acid binding protein, Antigen present cells, T lymphocytes, EAE

## Abstract

**Background:**

Multiple sclerosis (MS) is an autoimmune disease in which dysregulated immune cells attack myelin in the central nervous system (CNS), leading to irreversible neuronal degeneration. Our previous studies have demonstrated that epidermal fatty acid binding protein (E-FABP), widely expressed in immune cells, in particular in dendritic cells (DCs) and T lymphocytes, fuels the overactive immune responses in the mouse model of experimental autoimmune encephalomyelitis (EAE).

**Methods:**

In the present study, we conducted an intensive computational docking analysis to identify novel E-FABP inhibitors for regulation of immune cell functions and for treatment of EAE.

**Results:**

We demonstrate that compound [2-(4-acetylphenoxy)-9,10-dimethoxy-6,7-dihydropyrimido[6,1-a]isoquinolin-4-one; designated as EI-03] bound to the lipid binding pocket of E-FABP and enhanced the expression of peroxisome proliferator-activating receptor (PPAR) γ. Further *in vitro* experiments showed that EI-03 regulated DC functions by inhibition of TNFα production while promoting IL-10 secretion. Moreover, EI-03 treatment counterregulated T cell balance by decreasing effector T cell differentiation (e.g. Th17, Th1) while increasing regulatory T cell development. Most importantly, mice treated with this newly identified compound exhibited reduced clinical symptoms of EAE in mouse models.

**Conclusions:**

Taken together, we have identified a new compound which displays a potential therapeutic benefit for treatment of MS by targeting E-FABP.

## Background

Multiple sclerosis (MS) continues to be a serious public health problem without a curative treatment. Although the development of MS is attributed to a combination of genetic and environmental factors, the exact cause of MS is not completely understood [1,2]. The goals of MS therapy are to modify the disease course and to manage relapses and symptoms. For example, finglolimod has been shown to reduce relapses of MS patients by preventing pathogenic lymphocyte infiltration into the central nervous systems (CNS) [3,4]. However, a substantial proportion of MS patients do not respond well to the currently available medications. Therefore, identification of new targets for management of MS is urgently needed.

Fatty acid binding proteins (FABPs) constitute a family of cytosolic proteins which exhibit distinct tissue specificity [5]. As lipid chaperones, FABPs can regulate cellular metabolism and function through enabling fatty acid distribution and coordinating their responses. Since fatty acids function both as energy sources and as signaling molecules, FABPs have been identified as central regulators of metabolic and inflammatory pathways [6-9] Using a mouse model of MS, experimental autoimmune encephalomyelitis (EAE), we have demonstrated that mice deficient of FABPs, in particular epidermal FABP (E-FABP), have protection from the development of EAE [10,11]. E-FABP deficient dendritic cells (DCs) are defective in producing proinflammatory cytokines and in promoting Th1 and Th17 responses [11]. Furthermore, we have shown that CD4+ T cells deficient for E-FABP exhibit increased expression of peroxisome proliferator-activating receptor γ (PPARγ), which suppressed Th17 differentiation while enhancing regulatory T cell (Treg) development [10]. Based on our previous studies, it has become clear that the protection against EAE exhibited by E-FABP-deficient mice is due to the E-FABP-deficient phenotype of the antigen presenting cells (APCs) as well as of the T cells, themselves. Thus, E-FABP represents a new therapeutic target for EAE treatment, and modulating E-FABP activity may provide an attractive strategy for MS management.

Recently, many studies have reported the development of specific inhibitors which can modify FABP functions [12-15]. For example, BMS309403, a small molecule inhibitor of adipose FABP (A-FABP), has been shown to treat atherosclerosis and type 2 diabetes in mouse models [16], suggesting that inhibition of FABP activity is an effective approach for treatment of inflammatory diseases and metabolic syndromes. Herein, we identified 2-(4-acetylphenoxy)-9,10-dimethoxy-6,7-dihydropyrimido[6,1-a]isoquinolin-4-one (i.e., EI-03) as a novel E-FABP inhibitor using a ligand docking computational method [17,18]. Experimental evidence further confirmed that EI-03 effectively inhibited E-FABP activity in both DCs and T cells and potently reduced EAE symptoms in mouse models.

## Methods

### Computational modeling for E-FABP inhibitors

The three-dimensional structure of E-FABP was obtained from Protein Data Bank (PDB ID 1B56). The crystal structure was processed using the Protein Preparation Wizard in Maestro v9.3. Hydrogens were added consistent with a pH of 7.0. All water molecules were removed. Then the structure was energy minimized with an RMSD cutoff of 0.3 Å. The receptor grid was created with the centroid of the crystal ligand as the center. Virtual screening was carried out using the program Glide v5.7 [19]. Flexible docking was performed with the standard precision (SP) mode. More than one million commercially available compounds were downloaded from ZINC database and screened for potential inhibitors of E-FABP. The top-ranked compounds were clustered into different classes based on similarity of chemical structures. Finally, 5 commercially available compounds with distinct structures were purchased from Interbio Screen Ltd for following experimental tests.

### E-FABP purification and thermal shift assays

Recombinant His_6_-tagged E-FABP was over-expressed and purified from BL21 (DE3) cells. Protein was purified by Ni-NTA resin. Additional purification steps included cleavage of His_6_-tag using TEV protease followed by ion-exchange chromatography. Complete removal of endogenous lipids was ensured by final purification on Lipidex-1000 column. Purified *apo* E-FABP protein was used to probe small molecule compounds in a fluorescence based thermal shift assay [20]. Reactions were set-up in PCR tubes in a 20 μl volume containing 10 μM E-FABP and 10× SYPRO Orange dye (Invitrogen) in 20 mM HEPES pH 7 and 150 mM NaCl, containing either test compounds or DMSO only controls. Tested compounds were added at increasing concentrations such that the DMSO concentration never exceeded 2%. PCR tubes were then sealed, centrifuged and heated from 25 to 95 degrees at a rate of 1 degree/min on 7500 Real-Time PCR machine (Applied Biosystems). Raw data analysis and curve fitting to calculate Tm values was performed as previously described [20].

### Generation of bone marrow-derived dendritic cells (BMDCs)

Femurs and tibias from 8- to 10-wk-old mice were flushed with PBS, supplemented with 2% FBS to collect the bone marrow. Red cells in the bone marrow were lysed with red cell lysis buffer (R&D systems). The bone marrow was washed in DPBS and plated in 100-mm tissue culture dishes with 5% FBS RPMI 1640 medium at 37°C/5% CO_2_ for 4 hours. Then the non-adherent cells were plated in 5% FBS RPMI 1640 medium with 20 ng/ml GM-CSF (R&D Systems). New 5% FBS RPMI 1640 medium with 20 ng/ml GM-CSF was added on day 2 and day 5. The cultured BMDCs were collected on day 7 for further experiments.

### Real-time PCR

For real-time PCR analyses, RNA was extracted from cells using RNeasy Mini Kit (Qiagen). cDNA synthesis was performed with QuantiTect Reverse Transcription Kit (Qiagen). Quantitative PCR was performed with SYBR® Green PCR Master Mix using ABI 7500 Real-Time PCR Systems (Applied Biosystems). PPARβ/δ, PPARγ, TNFα, IL-10 and β-actin expression was analyzed by QuantiTect primer assays (Qiagen). Results were normalized to β-actin. Relative expression of the target genes was measured using the ΔΔCT approach.

### Confocal analysis

BMDCs cultured on poly-D-lysine coated coverslips (NeuVitro) in a 24-well plate were treated with EI-03 (10 μM) or DMSO control for 18 hours. After fixation and permeabilization, the cells were stained with anti-PPARγ antibody (EMD Millipore). Nuclei of BMDCs were stained with DAPI. Confocal analysis was performed with Nikon Eclipse TE2000 confocal microscopy.

### ELISA

1 × 10^6^ BMDCs were stimulated with Mtb (50 μg/ml) for 6 hours in the presence or absence of indicated concentrations of EI-03. Culture supernatants were collected for measurement of protein levels of TNFα and IL-10 with mouse ELISA kits (Biolegend) according to manufacturer’s instructions.

### T cell culture *in vitro*

Mouse naïve CD4^+^ T cells or CD8^+^ T cells were separated with a BD FACS Aria II Cell Sorter. Naïve CD4^+^ or CD8^+^ T cells were cultured with stimulation of anti-CD3 (5 μg/ml) and anti-CD28 (2 μg/ml). Th1 or CTL cells were differentiated with IL-12 (10 ng/ml); Th17 were induced with IL-6 (20 ng/ml) and TGFβ (1 ng/ml); Tregs were induced with TGFβ (1 ng/ml). In some experiments, cells isolated from spleen or lymph nodes were stimulated with anti-CD3/CD28 in the presence or absence of EI-03 (10 μM) for measurement of IFNγ production or Foxp3 expression in CD4^+^ T cells.

### Flow cytometric and cell sorting

Immune cells from draining lymph nodes or spleens were subjected to surface staining or cultured with PMA (5 ng/ml; Sigma), ionomycin (500 ng/ml; Sigma) and Golgiplug (BD) for 6 hours, then harvested for surface and intracellular staining. Flow cytometric data were collected with a BD FACS Calibur™. Flow cytometric data analyses were performed with Flowjo (Tree Star). The following antibodies were used for cell staining: anti-CD4 (clone RM4-5), anti-CD8 (clone 53–6.7), anti-MHC class II (clone M5/114.15.2), anti-IFN-g (clone XMG1.2), anti-IL-17 (clone TC11-18H10.1), anti-Foxp3 (clone FJK-16 s), anti-CD80 (clone 16-10A1) and anti-CD86 (clone GL-1).

### Mice and the EAE model

C57BL/6 mice were bred and maintained in the animal facility in the Hormel Institute in accordance with the University of Minnesota Institutional Animal Care and Use Committee (IACUC). Six to ten-week-old female mice were utilized for experiments. All animal protocols were approved by IACUC in the University of Minnesota and followed national guidelines. EAE was induced as previously described [10]. Briefly, Mice were injected in the flank with a 100 μl of emulsion containing 100 μg of MOG_35–55_ in CFA (Sigma-Aldrich) supplemented with 500 ng of *Mycobacterium tuberculosis* (Mtb) H37Ra (Difco Laboratories). Mice were injected i.p. with 200 ng of pertussis toxin (List Biological Laboratories) immediately following MOG_35–55_ injection (day 0) and again 2 days post immunization. For treatment with EI-03, EI-03 (10 mg/kg or 20 mg/kg) in 200 μl PBS was injected into mice by i.p. from day 0 or day 8 and injected every two days till day 20, the same volume of DMSO in PBS was injected into mice by i.p. as control. Clinical scores were designated numerically according to the following: 0, no detectable EAE symptoms; 1, tail paralysis/loss of tonicity; 2, abnormal gait; 3, hind limb paralysis; 4, hind limb and forelimb paralysis; and 5, moribund or dead; 0.5 gradations were assigned for intermediate scores.

### Isolation of mononuclear cells from mouse CNS

Brain and spinal cord of EAE mice were removed and cut into small pieces in a 70 mm cell strainer placed in a 10 cm petri dish containing 10 ml of ice-cold PBS. Each piece of the organ was pressed through the cell strainer using the back of a sterile 1 ml syringe plunger. After several washes of the cell-strainer, all collected cell suspensions were combined and centrifuged for 10 min at 400 g. The cells were resuspended with 7 ml PBS + 30% Percoll and overlay onto 3 ml of PBS + 70% Percoll and centrifuged at 800 g for 20 min at room temperature. The fat on the top of the tube was removed and the cells from the interface were collected and washed twice with PBS.

### Statistical analysis

All quantitative data were shown as means ± SD. Unpaired, two-tailed Student’s *t*-test was performed for comparison of results from different treatments. P value less than 0.05 is considered statistically significant.

## Results

### *In-silico* screening of potential E-FABP inhibitors

To identify novel E-FABP inhibitors, an intensive computer-aided molecular docking analysis using Glide v5.7 [19] was performed to screen 1 million drug-like compounds in chemical and natural compound libraries based on the crystal structure of E-FABP. First, an initial rough positioning and scoring phase was followed by torsionally flexible energy optimization on an OPLS-AA non-bonded potential grid for a few hundred surviving candidate poses. Next, the selected candidates with glide score less than −5.0 were refined *via* a Monte Carlo sampling of pose conformation and further evaluated using Glide XP (XP) mode for glide score and glide energy. Finally, the very best candidates were clustered using Canvas and top 10 representative compounds with diverse chemical structures were identified as the most promising E-FABP inhibitors (EI). As listed in Table 1, 5 commercially available compounds were purchased and tested experimentally for functional analyses using both *in vitro* and *in vivo* assays.

**Table 1:** **In**-**silico screening of potencial E-FABP inhibitors**

**No.**	**Molecular formula**	**Molecular weight**	**IUPAC name**
EI-01	C22H29N3O7	447.48816	benzyl 2-(((R-methoxy-2-oxoethyl)amino)-4-methyl-1-oxopentan-2-yl)carbamoyl)-5-oxopyrrolidine-1-carboxylate
EI-02	C20H18N4O4	378.38732	1-phenyl-6-(3,4,5-trimethoxyphenyl)-1H-pyrazolo[3,4-d]pyrimidin-4(5H)-one
EI-03	C22H20N2O5	392.4112	2-(4-acetylphenoxy)-9,10-dimethoxy-6,7-dihydro[6,1-a]isoquinolin-4-one
EI-04	C20H14N4O	326.35736	2-(furan-2-yl)-1-(m-tolyl)-1H-imi-dazo[4,5-b]quinoxaline
EI-06	C20H19NO5	353.37456	2-((−hydroxy-4-oxo-2-phenyl-4H-chromen-7-yl)oxy)-N-propylacetamide

### Molecular docking model of E-FABP/Inhibitor interactions

**Figure 1:**
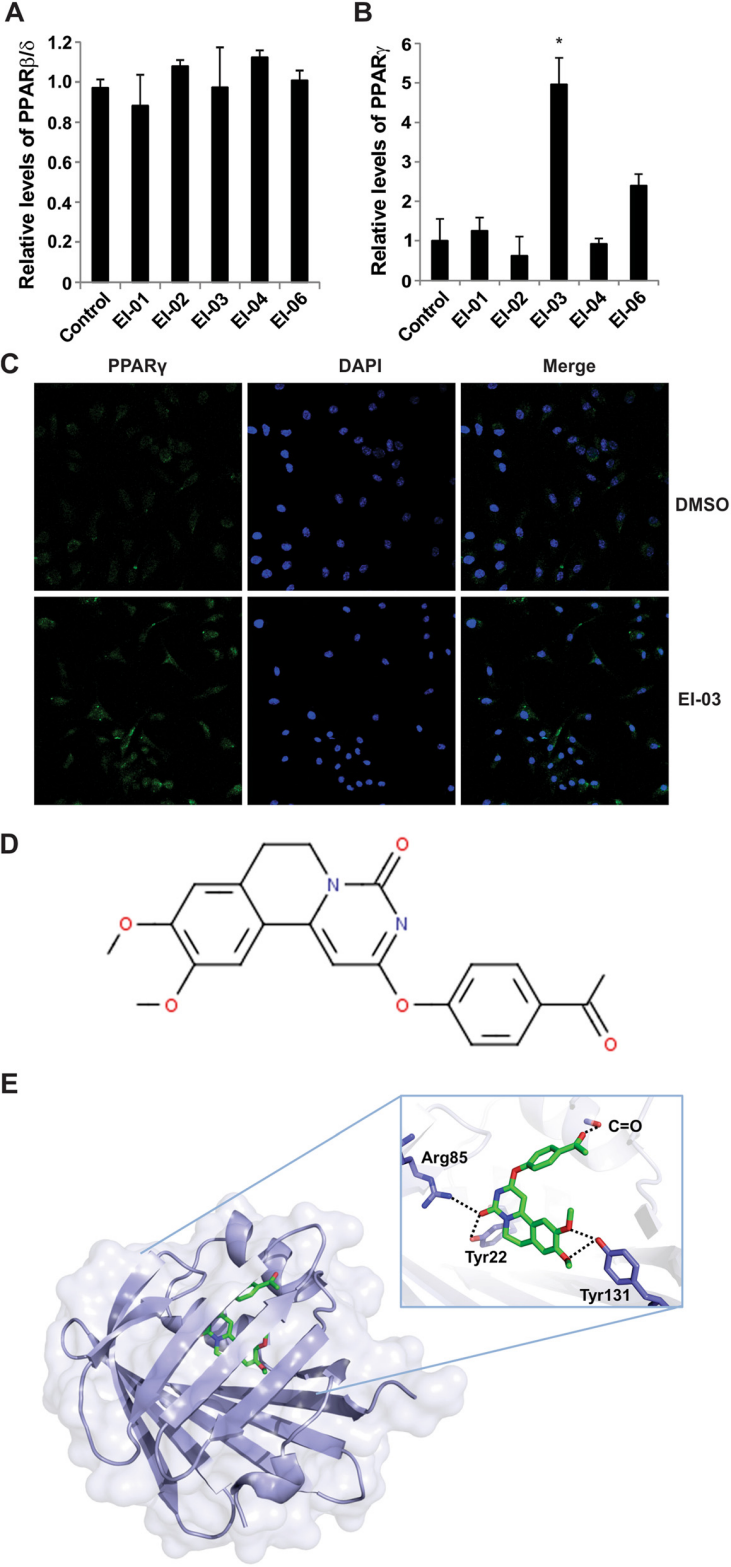
Screening of E-FABP inhibitors. T cells were isolated and incubated with potential E-FABP inhibitors (10 μM) or DMSO control for overnight. Expression of PPARβ/δ **(A)** and PPARγ **(B)** was examined by realtime-PCR (*, p < 0.05 as compared to the DMSO control). **(C)** Confocal analysis of PPARγ protein expression in BMDCs cultured either with DMSO or with EI-03 (10 μM) for 18 hours (green: PPARγ, blue: nuclei). **(D)** Chemical structure of the EI-03 (2-(4-acetylphenoxy)-9,10-dimethoxy-6,7-dihydropyrimido[6,1-a]isoquinolin-4-one). **(E)** Analysis of EI-03/E-FABP complex by a computational model. The protein was shown as a ribbon diagram and a partially transparent surface representation while the docked compound is shown as a ball-and-stick model. The inset shows a zoomed-in view of the main interactions highlighted by a network of hydrogen bonds.

In our previous studies, we demonstrated that E-FABP deficient T cells exhibit elevated expression of PPARγ, but E-FABP deficiency has no impact on PARβ/δ expression [10]. Thus, we tested all the potential E-FABP inhibitors by measuring alterations of PPAR expression in T cells. While all the tested inhibitors displayed minimal impact on PARβ/δ expression (Figure 1A), EI-03 remarkably enhanced PPARγ expression in CD4^+^ T cells and BMDCs (Figure 1B, C). These data suggested that EI-03 (see detailed structure in Figure 1C) is able to functionally inhibit E-FABP activity. We next analyzed the binding characteristics of EI-03 with E-FABP obtained from the molecular docking studies (Figure 1D). In this model, the binding pocket of E-FABP remained virtually unaltered and the EI-03 adopted the U-shaped conformation similar to what has been observed in the previous complex structures with fatty acid ligands (PDB access code 4LKT). The main E-FABP/EI-03 interactions consisted of five hydrogen bonds with the side chains of Tyr22, Arg81, and Try131 and backbone carbonyl oxygen of Met35 as well as van der Waals interactions with the surrounding hydrophobic residues. Tyr131 made a bidentate hydrogen bonding interactions with O4 and O5 of the inhibitor, maintaining the same interaction with the polar head group of the natural fatty acid ligands. Altogether, our data indicate that EI-03 displayed an optimal fitting with favorable interactions around the lipid binding pocket of E-FABP.

### EI-03 binds directly to E-FABP

**Figure 2:**
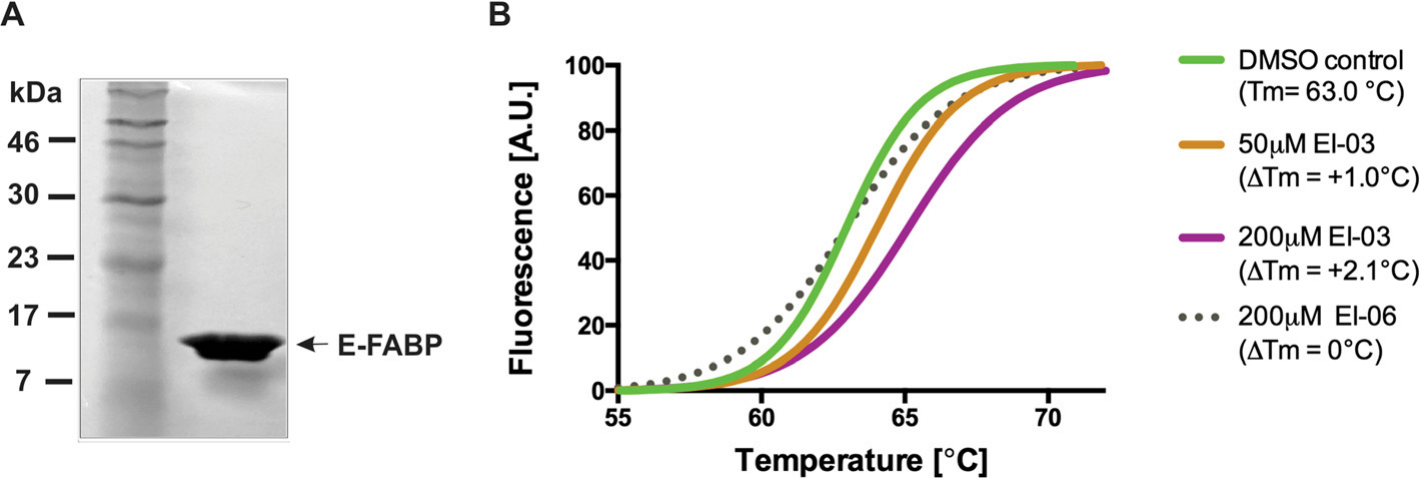
EI-03 binds directly to E-FABP. **(A)** A coomasie-stained gel depicting the purity of E-recombinant FABP protein (15.3 kDa) used in thermal shift assays. **(B)** Normalized melting curves depicting enhanced thermal stability of E-FABP in the presence of EI-03, as is evident by a Tm shift of 1.0-2.1°C when compared to EI-06 or DMSO control. The data shown are representative of three independent experiments.

On the basis of tight interactions of the EI-03/E-FABP complex indicated by the molecular docking model, we next performed an *in vitro* binding assay with the recombinant protein to confirm the direct binding of EI-03 to E-FABP. To obtain pure *apo*-proteins, residual endogenous ligands of E-FABP were removed by lipidex-chromatography (Figure 2A). The direct binding of EI-03 to E-FABP was measured by the thermal shift assay [20]. In this assay, the thermal stability of E-FABP was monitored by measuring the fluorescent signal of Sypro Orange in the presence or absence of the potential ligands. As shown in Figure 2B, EI-03 binding to E-FABP increased the temperature of unfolding of E-FABP (Tm) in a dose-dependent manner. In contrast, there was no change of Tm in the presence of compound EI-06 under the same conditions, suggesting a specific binding of EI-03 to E-FABP. Consistent with the molecular modeling results, these data further indicate that EI-03 specifically binds to E-FABP and thus inhibits its lipid-binding activity.

### EI-03 regulates cytokine production by DCs

**Figure 3:**
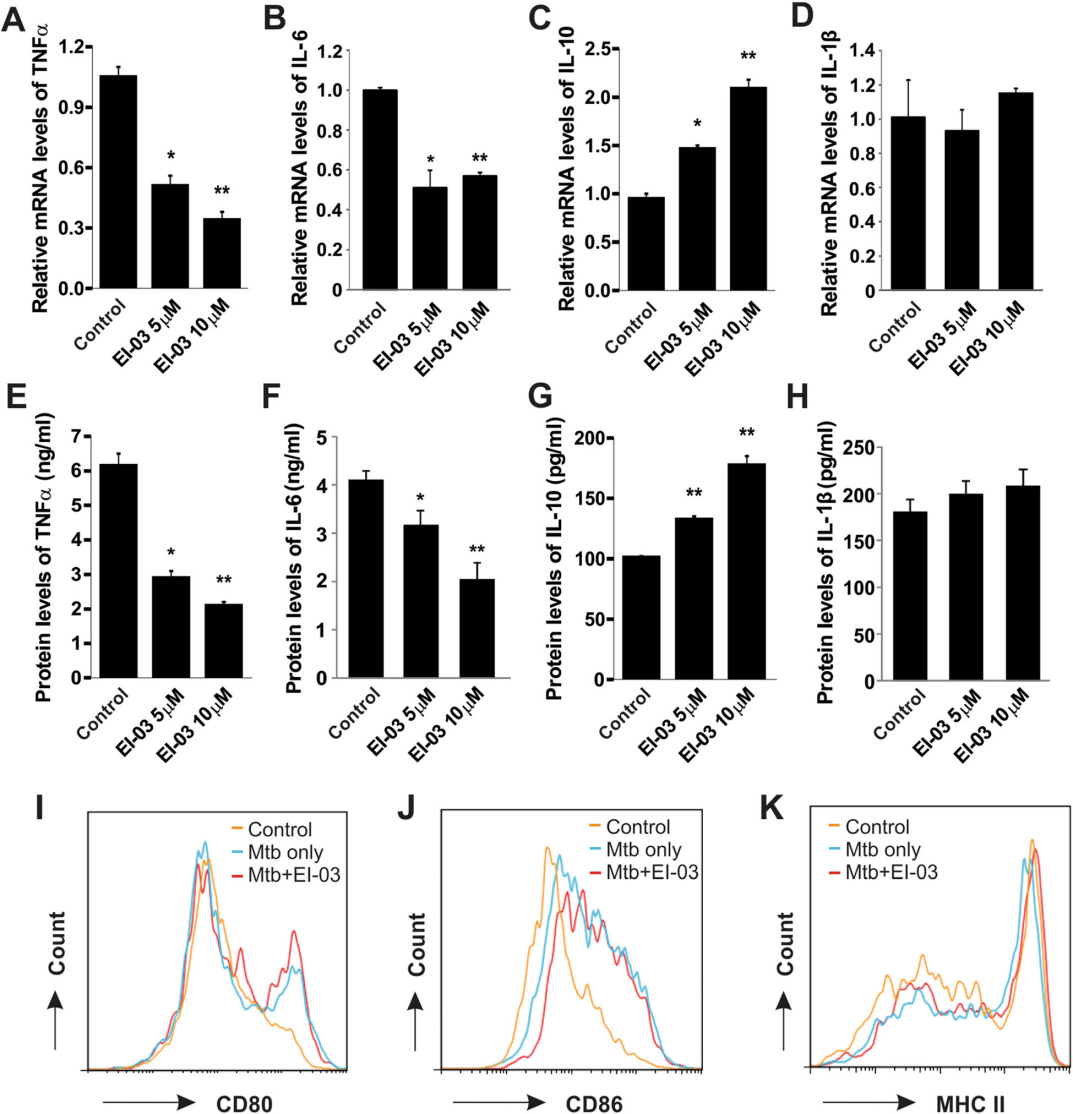
EI-03 regulates cytokine production by DCs. **(A-H)** Bone-marrow derived DCs were stimulated with Mtb (50 μg/ml) for 6 hours with the indicated concentrations of EI-03 or the DMSO control. Cells were lyzed for RNA extraction and cultural supernatants were collected for protein quantification. Relative mRNA levels of TNFα **(A)**, IL-6 **(B)**, IL-10 **(C)** and IL-1β **(D)** were measured by real-time PCR. Protein levels of each cytokine **(E-H)** were quantitated by ELISA (*, p < 0.05; **, p < 0.01 as compared to the DMSO control). For measuring expression of co-stimulatory molecules and MHCII expression, Mtb-stimulated DCs with or without EI-03 were analyzed for CD80 **(I)**, CD86 **(J)** and MHC II **(K)** levels with flow cytometry with respective antibodies. The results shown are representative of three independent experiments.

We have shown that E-FABP-deficient DCs exhibited reduced production of proinflammatory cytokines, but increased production of anti-inflammatory cytokines [11]. To this end, we examined whether DCs with inhibition of E-FABP activity by EI-03 mimicked the phenotype of E-FABP-deficient DCs. First, we determined the effects of EI-03 on cytokine production by DCs stimulated with Mtb, the most common adjuvant used in EAE models. EI-03 treatment significantly suppressed TNFα and IL-6 production by DCs at the transcriptional levels (Figure 3A, B). In contrast, mRNA levels of IL-10, but not IL-1β, by DCs was greatly elevated in the presence of EI-03 (Figure 3C, D). When we collected the cultural supernatants and measured each cytokine by ELISA, we confirmed above effects of EI-03 treatment of DCs at the protein levels (Figure 3E-H). We next analyzed the effects of EI-03 on expression of surface molecules which were critical for Ag presentation on DCs. As shown in Figure 3I-K, in response to Mtb stimulation, CD80, CD86, and MHC class II molecules were greatly upregulated. However, EI-03 treatment had no impact on the expression of MHC class II, CD80 and CD86 on Mtb-stimulated DCs, suggesting a minimal effect of EI-03 on antigen presentation. Therefore, EI-03 may regulate the function of antigen present cells by controlling the balance of cytokine production.

### EI-03 inhibits the differentiation of effector T cells

**Figure 4:**
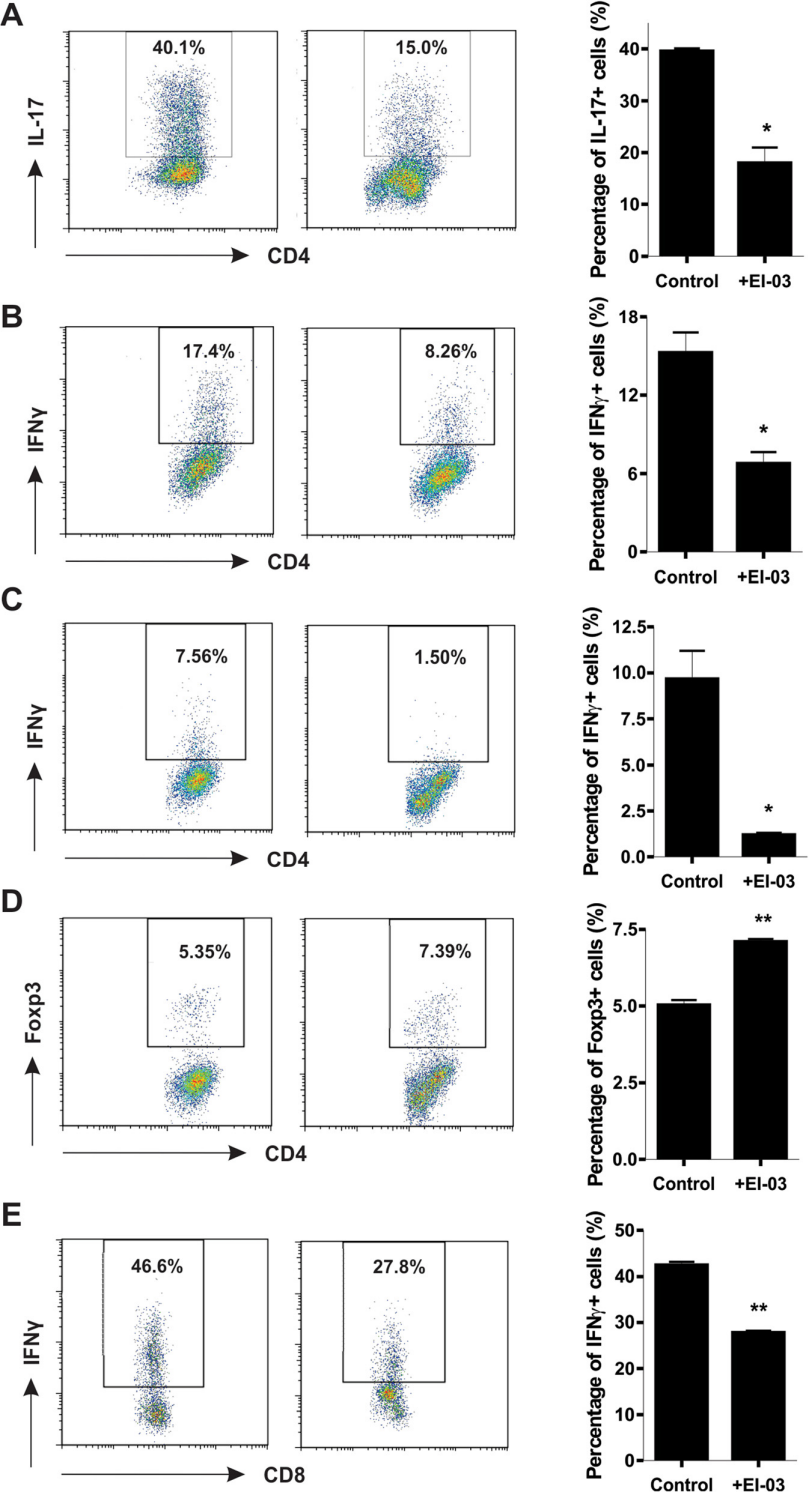
EI-03 regulates T cell differentiation. Naïve CD4^+^ T cells were differentiated into Th17 with IL-6 (20 ng/ml) and TGFβ (1 ng/ml) or Th1 with IL-12 (10 ng/ml) for 3 days in the presence or absence of EI-03 (10 μM). Production of IL-17 **(A)** and IFNγ **(B)** was measured by intracellular flow staining. Lymphocytes from LNs were cultured with anti-CD3/CD28 antibody in the presence or absence of EI-03 (10 μM) for 3 days. Foxp3 **(C)** and IFNγ **(D)** in CD4^+^ T cells were examined by intracellular staining. **(E)** Naïve CD8^+^ T cells were differentiated with IL-12 (20 ng/ml) in the presence or absence of EI-03 (10 μM) for 3 days. IFNγ production was examined by intracellular flow staining. Each panel displays a representative of three experiments yielding similar results. Data are presented as the means ± SD.

As E-FABP expression in T cells promotes Th17 differentiation [10], we reasoned that inhibition of E-FABP with EI-03 suppressed the production of IL-17 by CD4^+^ T cells. To this end, we differentiated naïve CD4^+^ T cells into Th17 cells with TGF-β/IL-6 in the presence or absence of EI-03. Indeed, EI-03 treatment significantly inhibited Th17 differentiation *in vitro* (Figure 4A). Of note, when we induced naïve T cells to Th1 cells *in vitro*, IFNγ production in Th1 cells was significantly reduced (Figure 4B). To further confirm this observation, we directly cultured lymphocytes from lymph nodes (LNs) with anti-CD3/CD28 antibodies in the presence of EI-03, and showed that IFNγ production from CD4^+^ T cells was also significantly inhibited by EI-03 treatment (Figure 4C). In contrast, Foxp3^+^ Tregs were upregulated with treatment of EI-03 (Figure 4D), which was consistent with what we observed in our previous studies [10]. Interestingly, CD8^+^ T cells from LNs also exhibited impaired IFNγ production in response to EI-03 treatment (Figure 4E). Moreover, cells isolated from spleen showed similar phenotypes in the presence of EI-03 (data not shown). Thus, our data demonstrated that EI-03 treatment counter-regulates the balance of effector T cells and Tregs *in vitro*. Due to the pathogenic roles of Th1 and Th17 cells and protective roles of Tregs in EAE development [21,22], these results imply that EI-03 could potentially inhibit EAE development through suppressing effector T cells and promoting Tregs, thereby representing a good drug candidate for EAE treatment.

### EI-03 treatment ameliorates EAE symptoms *in vivo*

**Figure 5:**
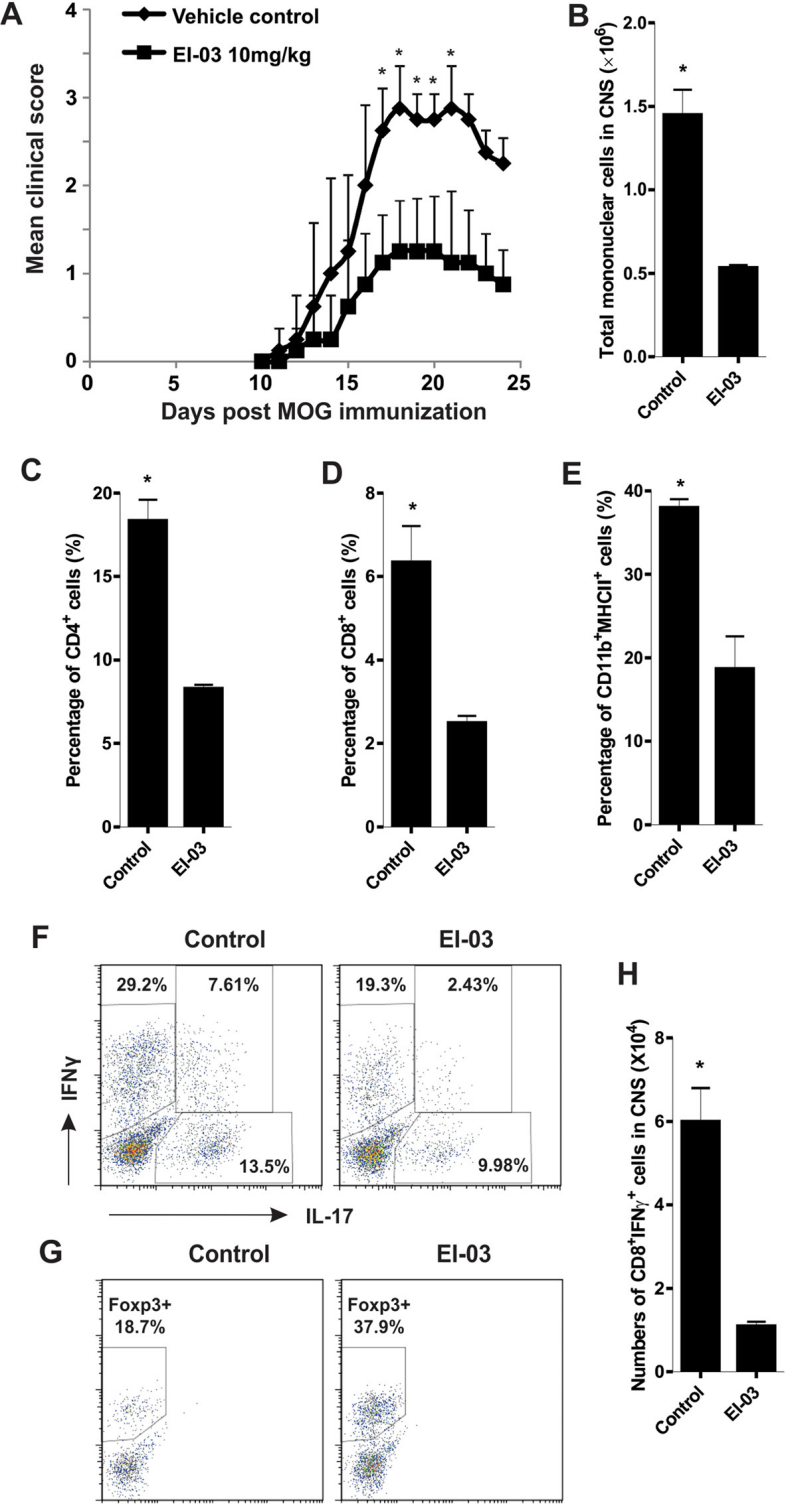
Amelioration of EAE is associated with reduced leucocyte infiltration and effector T cell function in EI-03-treated mice. **(A)** C57B/6 mice treated with EI-03 or DMSO vehicle control (n = 9/group) were immunized with MOG_35–55_ and scored daily postimmunization (*, p < 0.05). **(B)** EAE mice from EI-03-treated or control mice were sacrificed at the peak of the disease and total numbers of mononuclear cells in the CNS of those mice were measured with an automatic cell-counter. Percentage of infiltrated CD4^+^ T cells **(C)**, CD8^+^ T cells **(D)** and CD11b^+^MHCII^+^ cells **(E)** were analyzed by flow cytometric staining. The expression of IL-17, IFNγ **(F)** and Foxp3 **(G)** in CNS-infiltrated CD4^+^ T cells were determined using intracellular staining. **(H)** Total numbers of IFNγ^+^ CD8^+^ T cells in CNS were measured using intracellular staining. The results shown are representative of three independent experiments (*, p < 0.05).

Given the evidence that EI-03 regulated the functions of both APCs and T cells, suggesting a favorable outcome for treatment of autoimmune diseases, we evaluated the therapeutic efficacy of EI-03 using EAE models *in vivo*. We treated mice with EI-03 concurrent with MOG immunization on day 0 of the EAE model. We found that EI-03 treatment significantly ameliorated the severity of EAE disease with reduced clinical scores (Figure 5A). Further analyses showed that mice treated with EI-03 exhibited reduced numbers of total infiltrated leukocytes, into the CNS as compared to mice treated with vehicle control (Figure 5B). More specifically, the percentage of CD4^+^ T cells, CD8^+^ T cells and CD11b^+^MHCII^+^ populations in EI-03 treated mice was significantly lower than those in control mice (Figure 5C-E). Consistent with the *in vitro* data, CD4^+^ T cells in the CNS of EI-03-treated mice exhibited reduced production of IL-17 and IFNγ, but elevated expression of Foxp3, when compared to those from control mice (Figure 5F, G). In addition, CD8^+^ T cells in the CNS also displayed reduced expression of IFNγ in EI-03 treated mice (Figure 5H).

**Figure 6:**
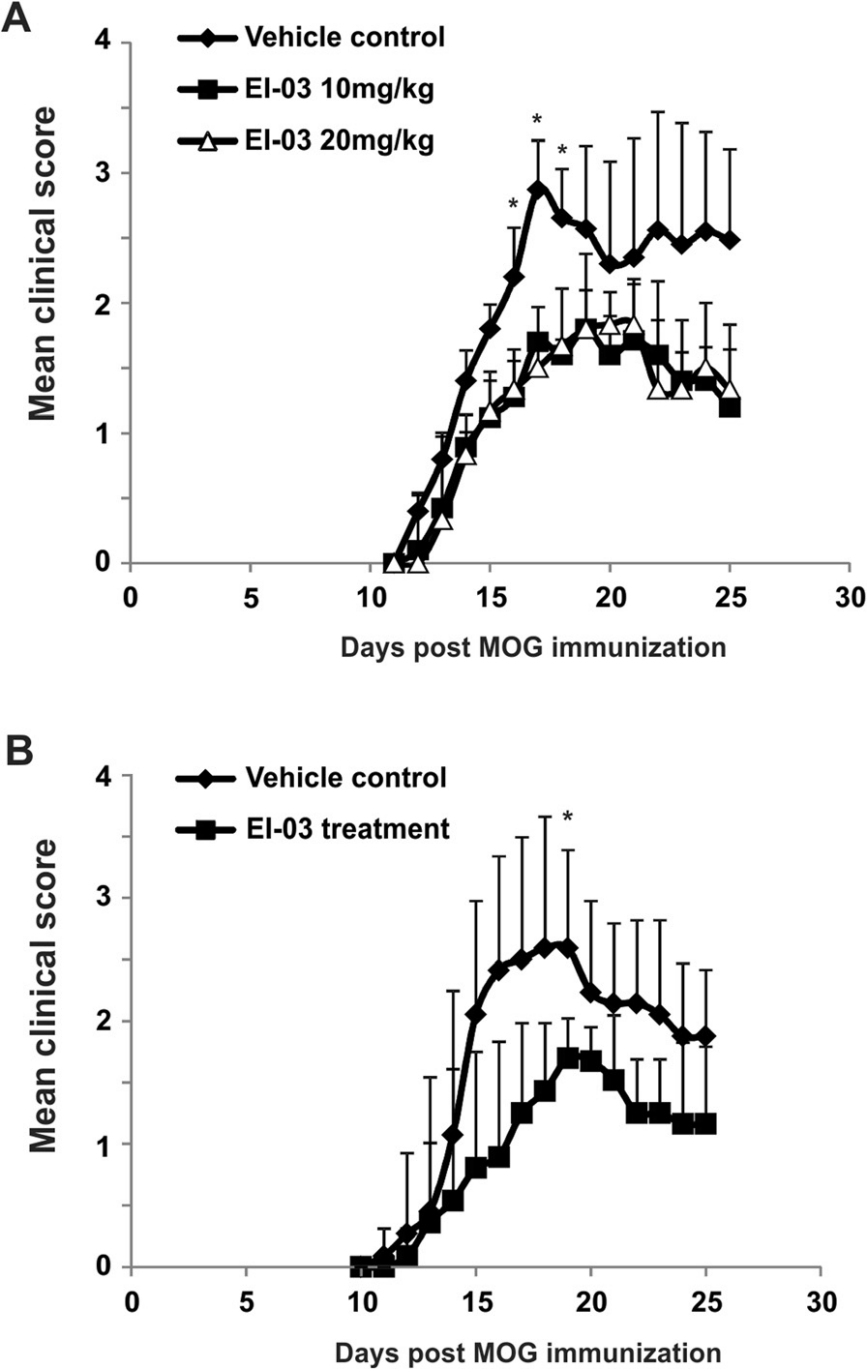
EI-03 treatment inhibits EAE disease. **(A)** Different dosages of EI-03 were administered to mice at the beginning of EAE induction with MOG immunization (n = 9/group). Vehicle DMSO was administered as non-treated controls. Clinical scores were measured daily (*, p < 0.05). **(B)** EI-03 compounds (10 mg/kg) were administered to mice on day 8 after EAE induction with MOG (n = 9/group). Average clinical scores were shown in panel B (*, p < 0.05).

Notably, when we treated mice with different dosages of EI-03, we found that there was no additional benefit at a higher dosage (20 mg/kg) of EI-03 (Figure 6A), suggesting that an effective efficacy of EI-03 treatment for EAE development can be achieved at a relative lower dosage (10 mg/kg). As MS treatment is usually initiated after the disease has been active and diagnosed, we further investigated whether administration of EI-03 was effective for treating active EAE. We started EI-03 treatment when mice began to show symptoms of EAE. As shown in Figure 6B, EI-03 treatment was able to effectively inhibit EAE symptoms even when administered during active disease. Taken together, our results indicate that EI-03 is effective in suppressing EAE development and progression, thus representing a promising drug candidate for treatment of patients with MS.

## Discussion

MS is an autoimmune disease of the CNS in which axons are demyelinated due to an attack of activated immune cells. Although many cell types, including oligodendrocytes, epithelial cells and microglial cells, are involved in the pathogenesis of MS, the crosstalk between innate APCs and autoreactive lymphocytes plays a central role in the initiation and progression of autoimmune responses. While MS is traditionally thought to be a Th1-cell mediated autoimmune disease, characterized by IFNγ inflammation, recent work has demonstrated that other T cell subsets also contribute to the development of MS. For example, Th17 cells can produce IL-17 and IL-22 to disrupt blood brain barrier (BBB) and induce neuronal dysfunction in the CNS [23,24]; CD8^+^ T cells have also been shown to be critical for the pathogenesis of MS [25]. In contrast, these effector T cell-mediated immune responses are normally suppressed by Tregs, which are able to maintain immune homeostasis of CNS immunity [26]. Thus, MS is associated with unbalanced T cell subsets marked by increased frequency of effector T cells and decreased Treg cells. As MS is a heterogeneous disease involving various factors and numerous pathogenic cells, treatments targeting a single mediator or one specific cell type may not be optimal for treatment of MS patients. Herein, we provide evidence that targeting E-FABP, a lipid carrier expressed abundantly in immune cells, represents a novel strategy for modulating the functions of DCs and T cells for the management of EAE.

In our previous studies, we have demonstrated that E-FABP expression in CD4^+^ T cells regulates T cell differentiation and function [10]. In addition, E-FABP is also highly expressed in DCs, promoting proinflammatory cytokine production. During the development of EAE, E-FABP is further upregulated in the CNS, contributing to EAE pathogenesis through promoting IL-17 and IFNγ inflammation [11]. Thus, E-FABP overexpression in immune cells may dysregulate their functions to promote autoreactivity for the development of autoimmune diseases. By taking advantage of the efficient molecular docking methods [19], we performed a systematic search of more than 1 million drug-like compounds. Based on the ranking of the conformational, orientational and positional space of the docked compounds, we selected the very best candidates predicted to bind the lipid binding pocket of E-FABP and experimentally tested their functions with both *in vitro* and *in vivo* models. As E-FABP regulates T cell differentiation through impacting nuclear receptor PPARγ expression/activity [10], we first investigated whether these compounds actively enhanced PPARγ expression. Indeed, we showed that EI-03 acted like an inhibitor of E-FABP in that it significantly increased PPARγ expression, mimicking the heightened PPARγ levels observed in E-FABP deficient T cells. More importantly, we showed that EI-03 was able to directly bind to the lipid binding pocket of E-FABP (Figures 1D and 2)*,* thus functioning as a specific inhibitor of E-FABP.

To examine the effect of EI-03 on APC functions, we found that EI-03 potently inhibited proinflammatory cytokine TNFα production by Mtb-treated DCs. In contrast, anti-inflammatory cytokine IL-10 was upregulated by EI-03 treatment. This phenotype is consistent with what we have seen in E-FABP deficient DCs. Since DCs also express A-FABP, although to a much less extent, we cannot exclude the possibility that EI-03 may bind to A-FABP contributing to the observed phenotype. As EI-03 treatment may impair the production of Th-differentiation related cytokines, such as IL-6, by DCs, we further analyzed the effect of EI-03 on T cell differentiation. We confirmed that EI-03 decreases IL-17 production while increasing Foxp3 expression in T cells, which corresponds to the effects observed in E-FABP deficient T cells. Notably, E-FABP is the predominant FABP member in T cells (unpublished data), it is reasonable to speculate that EI-03 may also regulate T cell differentiation through direct targeting E-FABP in T cells. In addition, EI-03 was also showed to inhibit IFNγ production both in CD4^+^ T cells and in CD8^+^ T cells, implying that EI-03 may exhibit a more broad effect in controlling the function of DCs and the differentiation of T cell subsets for treatment of T cell-mediated autoimmune diseases.

As studies of EAE have contributed to several approved MS medications [27], we used this model to further investigate the therapeutic efficacy of EI-03 *in vivo*. EI-03 administration significantly suppresses the clinical symptoms during EAE development (Figures 5 and 6). Further analysis showed that EI-03 treatment inhibited T cell migration into the CNS as well as the production of IL-17 and IFNγ of the infiltrated populations. Notably, when we titrated the dosage of EI-03 in mouse models, we did not observe any apparent toxicity of EI-03 even at the dose of 20 mg/kg/day, regarding overall health condition, total cell counts and apoptotic status of peripheral blood, and activation markers of immune cells (data not shown). Thus, EI-03 is well tolerated when applied *in vivo*. Since PPARγ agonists have been shown to reduce clinical signs of EAE [28], we cannot exclude the possibility that administration of EI-03 *in vivo* may directly activate other targets, including PPARγ to exert its protective effects on EAE development. In addition, it warrants further investigation as to whether EI-03 treatment affects functions of other populations besides DCs and T cells during EAE development.

## Conclusion

In summary, we have demonstrated that targeting E-FABP represents a novel strategy for treatment of EAE. Using a molecular docking model, we identified a new E-FABP inhibitor which can regulate functions of both APCs and T cells. Further analyses with animal models of MS revealed a therapeutic benefit of the inhibitor in ameliorating clinical symptoms of EAE through inhibition of lymphocyte migration and pathogenic functions. Thus, these data suggest that this newly-identified inhibitor may be a new drug candidate for treatment of MS and other autoimmune diseases.
